# Management of Coagulopathy in Bleeding Patients

**DOI:** 10.3390/jcm11010001

**Published:** 2021-12-21

**Authors:** Stefan Hofer, Christoph J. Schlimp, Sebastian Casu, Elisavet Grouzi

**Affiliations:** 1 Department of Anaesthesiology, Westpfalz-Klinikum Kaiserslautern, 67655 Kaiserlautern, Germany; 2 Department of Anaesthesiology and Intensive Care, AUVA Trauma Hospital Linz, 4010 Linz, Austria; christoph.schlimp@trauma.lbg.ac.at; 3 Ludwig Boltzmann Institute for Experimental and Clinical Traumatology, AUVA Research Center, 1200 Vienna, Austria; 4 Emergency Department, Asklepios Hospital Wandsbek, 22043 Hamburg, Germany; s.casu@asklepios.com; 5 Transfusion Service and Clinical Hemostasis, Saint Savvas Oncology Hospital, 115 22 Athens, Greece; grouzielisavet@gmail.com

**Keywords:** haemostasis, acquired coagulopathy, goal-directed coagulation management, coagulation factor concentrate, anticoagulation reversal, viscoelastic testing

## Abstract

Early recognition of coagulopathy is necessary for its prompt correction and successful management. Novel approaches, such as point-of-care testing (POC) and administration of coagulation factor concentrates (CFCs), aim to tailor the haemostatic therapy to each patient and thus reduce the risks of over- or under-transfusion. CFCs are an effective alternative to ratio-based transfusion therapies for the correction of different types of coagulopathies. In case of major bleeding or urgent surgery in patients treated with vitamin K antagonist anticoagulants, prothrombin complex concentrate (PCC) can effectively reverse the effects of the anticoagulant drug. Evidence for PCC effectiveness in the treatment of direct oral anticoagulants-associated bleeding is also increasing and PCC is recommended in guidelines as an alternative to specific reversal agents. In trauma-induced coagulopathy, fibrinogen concentrate is the preferred first-line treatment for hypofibrinogenaemia. Goal-directed coagulation management algorithms based on POC results provide guidance on how to adjust the treatment to the needs of the patient. When POC is not available, concentrate-based management can be guided by other parameters, such as blood gas analysis, thus providing an important alternative. Overall, tailored haemostatic therapies offer a more targeted approach to increase the concentration of coagulation factors in bleeding patients than traditional transfusion protocols.

## 1. Introduction

Acquired bleeding disorders are characterised by abnormal haemostasis and represent a major challenge for emergency room physicians. They can develop predominantly as a result of medical conditions, such as liver disease, traumatic injury, surgical procedures, medical interventions, or iatrogenic causes such as anticoagulation medications or massive transfusions [1]. Disrupted balance between coagulation and fibrinolysis leads to coagulopathy and is typically characterised by impaired clot formation leading to spontaneous, prolonged or excessive bleeding, as well as thrombotic tendency [2]. Coagulopathy might occur as a result of dysfunction, reduced levels or absence of coagulation factors, as well as dysfunction or reduced levels of platelets [2]. Deficiencies of coagulation factors may be induced acutely, for instance following the loss of a large amount of blood after major trauma or surgery, which would necessitate fluid replacement therapy and a variety of blood component therapies leading to haemodilution [3]. They may also develop over time, for example due to a vitamin K deficiency or chronic anticoagulation therapy to control hypercoagulability; both can be associated with bleeding in case of overdosing or vascular injury [4].

Early recognition of coagulopathy is crucial for its prompt and successful management and improved clinical outcomes after major blood loss. A variety of haemostatic management approaches have become available and are being increasingly applied into daily practice including point-of-care (POC) testing, plasma-derived, or recombinant coagulation factor concentrates (CFCs), such as fibrinogen concentrate (FC), prothrombin complex concentrate (PCC), factor XIII (FXIII) concentrate, leading to an approach tailored to patients’ needs.

These topics formed the basis of a symposium at the virtual 21st Annual NATA Symposium on Patient Blood Management, Haemostasis and Thrombosis. This review summarises key principles from the symposium, with the aim of determining optimal management strategies of patients with acquired coagulopathies.

## 2. Management of Acquired Bleeding Disorders

The extent of coagulopathy depends on numerous factors including severity of bleeding, hypothermia, acidosis and patient characteristics, such as body size, comorbidities, and medication (e.g., anticoagulants) [4]. Certain treatments such as fluid replacement with non-blood solutions used to maintain normovolemia or administration of blood products such as red blood cells (RBCs) can also cause haemodilution [5]. The main goals of haemostatic management are to treat hyperfibrinolysis early and to restore clot formation and strength, which depends on the interaction of several factors, including fibrin network, activated platelets and activated FXIII [6]. Coagulation factors do not decrease homogeneously in severe bleeding. Although there may initially even be a tendency towards increased thrombin generation, the substrate fibrinogen reaches critically low plasma concentrations at an earlier stage than other coagulation factors [7]. Fibrinogen, also known as coagulation factor I, is a blood plasma protein that plays a crucial role in the coagulation process and is also the most abundant of the coagulation factors [8]. Normal plasma fibrinogen concentrations range from 1.5–4.5 g/L [9,10,11]. Fibrinogen concentration of <1.5–2 g/L (hypofibrinogenaemia) is associated with an increased bleeding risk and thus needs to be addressed first in the haemostatic treatment [12]. Moreover, fibrinogen levels <1 g/L are a strong independent risk factor for death after injury in patients requiring a massive transfusion [13]. Recent European guidelines recommend fibrinogen threshold levels of 1.5–2 g/L relating to perioperative bleeding and trauma [12,14]. In pregnant women, normal levels of fibrinogen at delivery are higher than in non-pregnant women (3.5–6.5 g/L vs. 2.0–4.5 g/L), attributable to normal physiological changes in haemostasis during pregnancy creating a state of hypercoagulability [15,16]. For this reason, the exact fibrinogen threshold for intervention during postpartum haemorrhage (PPH) is unclear [14]. However, low fibrinogen levels (<2 g/L) during delivery are predictive of progression to severe PPH [17,18].

Protocol-based management of patients with massive haemorrhage often includes a massive transfusion protocol (MTP), i.e., rapid allogeneic blood-product based transfusions at a fixed ratio approximating those found in the whole blood [6]. The main purpose of administering RBCs, fresh frozen plasma (FFP), and platelets during MTP is to maintain physiological levels and prevent a deficit of blood constituents [19]. MTPs are largely empirical and used in acute haemostatic management before results of standard laboratory tests (SLTs) are available to guide coagulation factor replacement [19].

During haemostatic treatment, coagulation factors can be substituted by administering FFP, cryoprecipitate or specific factor concentrates, such as FC, PCC or FXIII concentrate. However, the variable range of fibrinogen levels found in plasma makes it difficult to ensure target fibrinogen levels are achieved when FFP is used and large volumes of FFP are required [20,21]. For example, a mathematical model of the relationship between plasma fibrinogen levels and the amount of transfused haemostatic agent showed that using FFP to raise baseline fibrinogen levels from, e.g., 0.75 g/L to target 1.75 g/L in a bleeding patient was not possible, as the required volume increases exponentially with the fibrinogen concentration in plasma approaching the target level [22]. Moreover, infusion of large FFP volumes exposes the patients to the risk of transfusion-associated circulatory overload (TACO) [23]. A study by Khan et al. reported that patients receiving mixed transfusion packages of FFP with RBCs with late or no fibrinogen supplementation did not improve in neither lactate levels, a prognostic marker of tissue hypoperfusion and haemorrhagic shock, nor any ROTEM parameters characterising the clot strength and clotting time (CT) [24]. Overall, more concentrated agents such as cryoprecipitate or CFCs (i.e., FC) appear to be superior to FFP as first-line treatment [22,25]. Poor efficacy of treatment with FFP in comparison with FC was confirmed also by results of a meta-analysis of patient outcomes in massive trauma or surgery [23]. In direct comparison FC was superior to FFP in more than half of the measured outcomes including reducing blood loss, need for allogeneic blood products and length of hospital stay [23]. Moreover, the use of FC intraoperatively was shown to reduce the need for FFP; patients in major vascular surgeries who received FC, compared with those who received FFP, did not require any further supplementation with blood components apart from RBCs [26]. European guidelines now suggest FC as the first-line treatment for major bleeding accompanied by hypofibrinogenaemia [12].

## 3. Bleeding in Patients on Oral Anticoagulants

Patients at elevated risk of arterial or venous thrombosis (e.g., after coronary stenting, primary prophylaxis of atrial fibrillation, secondary prevention of venous thromboembolism, etc.) require anticoagulation treatment with vitamin K antagonists (VKA) such as warfarin or with direct oral anticoagulants (DOACs) [27]. There has been an increasing trend towards anticoagulant therapy in primary prevention with particularly DOAC prescriptions rising rapidly [28,29]. However, in cases of major or life-threatening bleeding, urgent surgery, or sometimes at supratherapeutic levels of anticoagulant without bleeding, urgent reversal of the anticoagulant drug is required.

### 3.1. Treatment of VKA-Associated Bleeding

The treatment of bleeding related to VKA can be achieved by co-administering vitamin K with either FFP or PCC [30]. The treatment goal is to increase the concentration of vitamin K-dependent coagulation factors including factors II (FII), VII (FVII), IX (FIX), and X (FX) [30]. Vitamin K induces de novo synthesis of these factors in the liver; therefore, this treatment has a slow onset of action (4–6 h) and may be inadequate for acute haemorrhage treatment [30,31]. Plasma transfusion (FFP or thawed plasma) is commonly used to provide an immediate source of these factors, but to restore haemostatic levels, administration of a large volume is needed, which may lead to fluid overload [32].

PCC represents an alternative source of vitamin K-dependent coagulation factors; 3-factor PCC (3F-PCC) contains FII, FIX and FX whilst 4-factor PCC (4F-PCC) contains FII, FVII, FIX and FX, proteins C and S, and heparin [33]. The concentration of clotting factors in PCC is about 25 times higher than that in plasma, therefore, its administration reduces the risk of fluid overload [33]. The superiority of PCCs over plasma to treat VKA-associated bleeding was shown in several studies [34,35,36]. A meta-analysis of 13 studies comparing the use of PCCs and FFP for warfarin reversal showed that PCC was associated with a significant reduction in all-cause mortality [35]. PCC administration in comparison with FFP was also more likely to achieve normalisation of international normalised ratio (INR, odds ratio [OR] 10.80; 95% confidence interval [CI], 6.12–19.07) and the time to correction was shorter (mean difference −6.50 h; 95% CI, −9.75 to −3.24) [35]. Moreover, patients treated with PCC had a lower risk of TACO compared with FFP (OR 0.27; 95% CI, 0.13–0.58) and no significant difference in the risk of thromboembolism between patients receiving PCC or FFP was observed (OR 0.91; 95% CI, 0.44–1.89) [35]. A meta-analysis by Brekelman et al. showed that PCC was more effective than FFP in correcting INR in patients with bleeding complications within 1 h after administration (1.4–1.9 vs. 2.2–12, respectively) [34]. A recent meta-analysis including 17 studies with a total of 2606 participants reported similar findings [36]. Treatment with PCC in comparison with FFP decreased 90-day all-cause mortality, improved INR reversal and had a lower risk of adverse events [36].

One of the side effects of VKA treatment is a high risk of bleeding, particularly of intracerebral haemorrhage (ICH) [37]. Moreover, patients with VKA-associated ICH (VKA-ICH) are at higher risk of in-hospital haematoma expansion compared with non-VKA-treated patients [38]. A retrospective study comparing mortality in patients with VKA-ICH who received FFP and/or PCC suggested that reversal of VKA-ICH with a combination of FFP and PCC might be associated with the lowest mortality (27.8%), followed by PCC only (37%) and FFP only (45.6%) [39]. A randomised trial in patients with VKA-ICH showed faster INR normalisation with PCC than with FFP, which in turn was associated with smaller haematoma expansion [40]. A meta-analysis by Hill et al. showed that PCC treatment in patients with VKA-ICH resulted in lower mortality and improved INR reversal compared with FFP treatment [36]. Overall, the studies show superiority of PCC over FFP for urgent treatment of VKA-associated bleeding and its use has been now adopted by many guidelines, as summarised in Table 1 [12,14,41,42,43,44,45].

### 3.2. Treatment of DOAC-Associated Bleeding

DOACs are a newer class of oral anticoagulants that include direct activated FX (FXa) inhibitors (e.g., apixaban, edoxaban, and rivaroxaban), and the direct thrombin inhibitor, dabigatran. In comparison with VKAs, DOACs have the advantages of a rapid onset of action, within 2–3 h, and more predictable pharmacokinetics and pharmacodynamics [27,55]. Specific DOAC reversal agents such as andexanet alfa for apixaban and rivaroxaban reversal and idarucizumab for dabigatran reversal are currently available [56]. However, these specific antidotes, particularly andexanet alfa, have limitations including high cost, safety concerns and mechanistic limitations [56], or might not be widely available, which makes managing a life-threatening haemorrhage in a patient receiving FXa inhibitors challenging [55,57]. Furthermore, in emergency situations the administration of specific reversal agents can be delayed if the type and plasma level of DOAC present in a patient’s blood needs to be rapidly identified with specific tests [58,59]. Non-specific strategies for the treatment of DOAC-associated bleeding, such as administration of PCC, are now widely recognised and recommended by different organisations for situations when specific agents are not readily available [12,14,41,42,47,48,50,51,52,54,60]. The guideline recommendations are summarised in Table 1.

PCC can effectively restore haemostasis in patients requiring urgent treatment of bleeding related to DOACs as was shown in multiple clinical studies. The UPRATE study investigated the effectiveness of 4F-PCC to treat bleeding associated with FXa inhibitors in two cohorts (Sweden and Canada) and showed comparable haemostatic effectiveness in both groups (69% and 68% of patients, respectively); the authors suggested that 4F-PCC could become an effective and relatively affordable option for management of DOAC-associated bleeding [61,62]. Furthermore, a retrospective study of 4F-PCC in patients with major bleeding and a need for treatment of FXa inhibitor-associated bleeding reported excellent or good haemostasis in 89% of cases [63]. Of particular interest is a comparison of a subgroup of patients in this trial that met stricter inclusion criteria similar to those used in the specific antidote andexanet alfa (ANNEXA-4) trial [64]. The reported clinical success rate was comparable between these two studies (90% and 82% for 4F-PCC and andexanet alfa, respectively) [63,64]. Another recent observational study showed that a low-dose of 4F-PCC promoted haemostasis in 83.8% of bleeding patients receiving FXa inhibitors [65].

Several retrospective studies compared the haemostatic efficacy of PCC vs. specific reversal agent for the treatment of ICH in patients receiving DOACs. Low-dose 4F-PCC was successfully used to manage DOAC-associated ICH in 94.7% of treated patients [66]. A retrospective analysis of adult patients with FXa inhibitor-related ICH receiving andexanet alfa or 4F-PCC reported no difference in functional outcome or thrombotic events between the two groups [67]. In another retrospective study of FXa-associated ICH, excellent or good haemostatic efficacy was observed in 16/18 (89%) patients treated with andexanet alfa, compared with 6/10 (60%) patients treated with 4F-PCC [68]. However, no statistical comparison of the two study groups was performed due to the small number of patients [68]. Lastly, a retrospective review of patients who received 4F-PCC for the treatment of oral FXa inhibitor-related ICH reported excellent or good haemostatic efficacy in 22/32 (69%) of patients with ICH following treatment with 4F-PCC compared with 135/168 (80%) of patients with ICH in the ANNEXA-4 trial [69].

Overall, there is significant heterogeneity in reporting standards, methodology, and outcomes across these small, retrospective studies; therefore, these results should be interpreted with caution [70]. In a recent meta-analysis of 22 studies of FXa inhibitor-associated bleeding treatment, the overall rate of good to excellent haemostatic control with 4F-PCC was 76% [71]. This study also reported a higher rate of thrombotic complications with andexanet alfa in comparison with aPCC/4F-PCC (13% vs. 4%, respectively) [71]. Similarly, another systematic review and meta-analysis comparing the haemostatic effectiveness of andexanet alfa or PCC for acute treatment of FXa inhibitor-associated haemorrhage reported a mean effectiveness for andexanet alfa of 82% at 12 h and 71% at 24 h and for PCC of 88% at 12 h and 76% at 24 h; these differences were not statistically significant [72]. Lastly, meta-analysis by Jaspers et al. also confirmed similar effectiveness of PCC and andexanet alfa in treatment of bleeding patients using FXa inhibitors [73]. However, in both studies the rate of thrombotic events was lower for PCC compared with andexanet alfa [72,73].

## 4. Management of Trauma-Induced Coagulopathy

Uncontrolled bleeding is the major cause of preventable death in patients following trauma [74]. At least one in four of all bleeding trauma patients show signs of coagulopathy at hospital admission [75,76,77]. The pathophysiology of trauma-induced coagulopathy (TIC) is complex, but the dilution or consumption of clotting factors is the main contributing factor [74]. TIC is associated with an increased rate of massive transfusions and mortality [75,78].

Assessment of coagulopathy and the following bleeding management are often guided by SLTs including prothrombin time (PT), INR, partial thromboplastin time (PTT), and Clauss fibrinogen test. However, the usefulness of SLTs in assessing or managing coagulopathy has been questioned [79]. SLTs provide only limited information (i.e., time of coagulation and platelet count), are time-consuming and tend to be omitted in situations where rapid treatment is needed, such as in severe bleeding [79]. In contrast, POC testing with viscoelastic tests (VET) provides information in real time [80]. VET, e.g., thromboelastometry (ROTEM) and thrombelastography (TEG), measure mechanical properties of the clot formation and provide dynamic information on the speed of coagulation initiation, kinetics of clot growth, clot strength, and breakdown of the clot [81]. The advantages and disadvantages of SLTs and VET testing are summarised in Table 2.

In emergency situations, haemostatic treatment can be initiated with empirical fixed ratio-based transfusion protocols. However, as the haemostatic state of the patient is unknown, these protocols are in a sense ‘blind’ transfusions [83]. MTPs vary between hospitals, and the optimal mixture ratio of RBC:FFP:platelet concentrate (PC) is a matter of debate [81]. MTPs are associated with insufficient transfusion of both FFP and platelets, which may aggravate bleeding [83]. Analysis of an in-house transfusion algorithm with a fixed ratio of RBC:FFP:PC of 6:3:1 showed that 82% patients received “insufficient” amounts of FFP and platelets and these amounts were on average 50% lower compared with the calculated amounts [83]. Another study showed that reconstitution of whole blood in a 1:1:1 (RBC:FFP:PC) fixed ratio resulted in significantly lower coagulation factor activity, endogenous thrombin potential and abnormal standard coagulation tests, as well as reduced haematocrit and platelet count [84]. Moreover, fibrinogen concentration was found to be significantly lower in reconstituted blood compared with citrated whole blood [84].

In contrast to MTPs, goal-directed coagulation management (GDCM) algorithms are based on VET results and the treatment is adjusted to the actual needs of the individual patient [80]. Thanks to the short turnaround of VET testing (first results available within 10 min) it is possible to check the effectiveness of the treatment and re-adjust it accordingly at the bedside [6]. GDCM of trauma patients was shown to reduce mortality and to be more efficient than treatment with MTPs [24,85,86,87]. A retrospective analysis including trauma patients showed that the observed mortality in patients receiving GDCM guided by ROTEM was lower than predicted by the trauma injury severity score [86]. Another study comparing transfusion of FFP and CFCs as the first-line therapy for reversal of TIC was terminated early for futility and safety reasons [25]. FFP showed poor efficacy in limiting blood loss or correcting TIC, and a large proportion of patients required rescue therapy [25].

Since GDCM monitors the patient’s haemodynamic state with POC before and during treatment, it reduces the risks of overuse of blood products (especially RBC and FFP) as well as under-transfusion [88]. A comparison between two cohorts of trauma patients treated before and after the implementation of changes in trauma management protocols (i.e., before and after inclusion of GDCM) showed reduced incidence of massive transfusion and a reduction in the transfusion of RBC and FFP in GDCM protocols [88]. Consequently, GDCM with the use of FC instead of FFP reduced not only the use of allogeneic blood products but also the related costs [88]. Implementation of GDCM protocols including POC in two Italian trauma centres led to a reported cost reduction of 23% [89]. Overall, GDCM and use of CFCs (such as FC, PCC, FXIII) may reduce costs associated with transfusion of blood products in patients with acquired bleeding [12].

## 5. Goal-Directed Coagulation Management for TIC Patients

**Figure 1:**
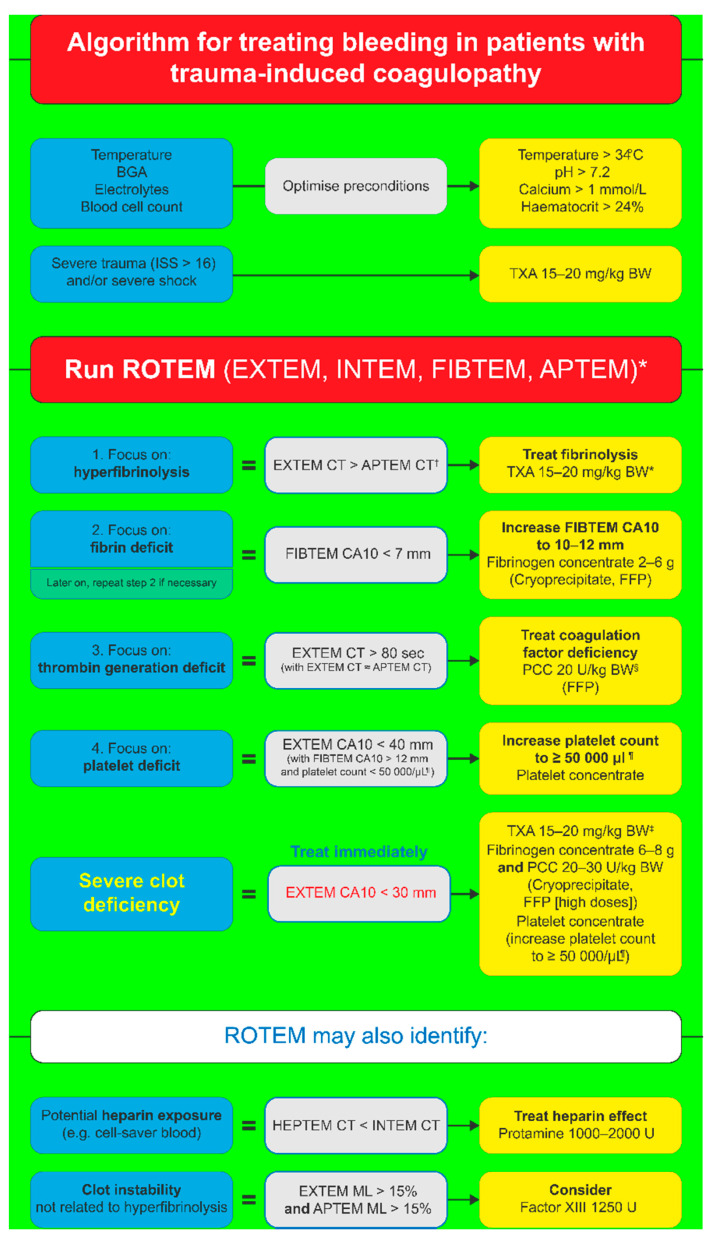
ROTEM-guided treatment algorithm for the management of trauma-induced coagulopathy. Adapted from Schochl et al., 2012 [6]. Reprinted from Springer Nature as indicated in the Terms and Conditions of the Creative Commons Attribution license. * For patients who are unconscious or known to be taking platelet inhibitor medication, Multiplate tests (adenosine diphosphate [ADP] test, arachidonic acid [ASPI] test, and thrombin receptor activating peptide-6 [TRAP] test) are also performed. ^†^ Any major improvement in APTEM parameters compared to corresponding EXTEM parameters may be interpreted as a sign of hyperfibrinolysis. ^‡^ Only for patients not receiving TXA at an earlier stage of the algorithm. ^§^ If decreased ATIII is suspected or known, consider co-administration of ATIII. ^¶^ Traumatic brain injury: platelet count 80,000–100,000/μL. CA_10_, clot amplitude at 10 min; BGA, blood gas analysis; BW, body weight; CT, clotting time; FFP, fresh frozen plasma; ISS, injury severity score; MCF, maximum clot firmness; ML, maximum lysis; PCC, prothrombin complex concentrate; TXA, tranexamic acid.

An example of ROTEM-guided haemostatic treatment algorithm for TIC is depicted in Figure 1. Individual steps are based on VET results, and the treatment utilises CFCs to restore clot strength, as explained below [6].

### 5.1. Fibrinogen Supplementation

Hypofibrinogenaemia as a result of blood loss, factor consumption, or haemodilution is associated with poor patient outcomes and increased mortality in trauma patients [90]. Fibrinogen concentration on arrival at the hospital may vary depending on individual patient factors; for instance, low fibrinogen levels have been associated with young age, male gender, long time elapsed since injury, low base excess, and high injury severity score [90]. Besides tranexamic acid, FC is the primary pharmacological intervention in TIC [91]. Fibrinogen plasma levels can be indirectly assessed by the FIBTEM test, a type of ROTEM analysis that examines the properties of fibrin-based clot independently by using extrinsic activator (tissue factor) and platelet inhibitor (cytochalasin D) [86]. This test has a high predictive value for massive transfusion in trauma patients comparable with SLTs [92]. FIBTEM amplitude at 10 min following the start of clot formation (FIBTEM_A10_) correlates with fibrinogen concentration [93], and, therefore, allows early identification of fibrinogen deficiency [94]. FIBTEM_A10_ < 7 mm has been suggested as a trigger for fibrinogen substitution with the aim to raise FIBTEM_A10_ to at least 10 mm in ongoing bleeding [6].

Fibrinogen supplementation may reduce transfusion requirements with corresponding reductions in morbidity and mortality. Recent studies showed that early FC administration (within one hour of admission) was associated with increased plasma fibrinogen concentration and a favourable survival rate among severe trauma patients [95,96]. The treatment of severe trauma patients with FC as the first-line therapy in bleeding management after hospital admission is unlikely to increase prothrombotic risk [91]. FC supplementation does not lead to higher fibrinogen levels post-trauma (>24 h after treatment) [91].

### 5.2. Thrombin Generation

In TIC, endogenous thrombin levels remain relatively stable despite dilution [97]. Thrombin generation is higher in trauma patients than in healthy controls as a result of haemostatic dysregulation and systemic changes [98]. During bleeding management, thrombin generation deficit should be considered only after the fibrinogen levels are adjusted [99]. FC supplementation to reach the therapeutic goal of FIBTEM_A10_ > 10–12 mm also often reduces CT to normal levels [14,99]. A recent in vitro study also showed that the substitution of PCC was not necessary to support clot formation and sufficient thrombin generation in case of sufficient fibrinogen supplementation following dilution [97]. Fibrinogen substitution, however, played a key role in the preservation of haemostatic capacity [97]. However, if EXTEM CT remains > 80 s after FC administration, thrombin generation can be improved by PCC supplementation [6]. There is limited evidence evaluating PCC as first-line therapy in trauma-associated bleeding and further studies are needed to assess its efficacy and safety [100].

### 5.3. Platelet Supplementation

Platelets play a key role in haemostasis and clot formation. Although very few trauma patients have low platelet numbers on admission [101], platelet deficit will very likely develop over time depending on the treatment [14]. Insufficient number of platelets is characterized by EXTEM_CA10_ < 40 mm (but normal FIBTEM amplitude) and low platelet count (<50,000/µL), which will indicate the need for platelet concentrate administration [6].

### 5.4. Clot Stability and FXIII

FXIII is known to be an essential contributor to clot strength by its ability to crosslink and stabilise fibrin [102]. However, most bleeding management guidelines currently do not include measurement and subsequent supplementation of FXIII [103]. Clot instability due to FXIII deficiency has been identified in some cases by ROTEM [103,104]. In the neurosurgical setting, a postoperative FXIII level < 60% was found to be an independent risk factor for postoperative ICH [105]. In cases of bleeding and low FXIII activity (e.g., <30%), administration of FXIII concentrate (30 IU/kg) is suggested [12].

## 6. Goal-Directed Coagulation Management without POC Testing

**Figure 2:**
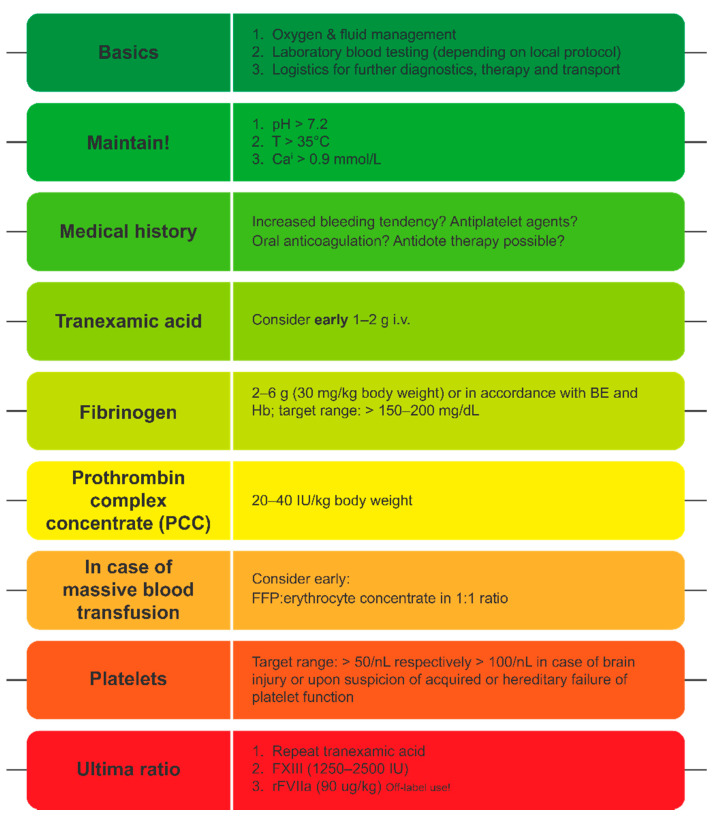
Simplified treatment algorithm for the initial assessment and management of trauma-induced coagulopathy without VET. Adapted from Casu S, 2021 [107]. Reprinted by permission from BMJ as indicated in the Terms and Conditions of the Creative Commons CC-NY license. BE; base excess; Ca, calcium; FFP, fresh frozen plasma; FXIII, factor XIII; Hb, haemoglobin; INR, international normalised ratio; PC, platelet concentrate; pRBC, packed red blood cells; rFVIIa, activated recombinant factor VII; TBI, traumatic brain injury.

GDCM algorithms offer a systematic approach to the management of bleeding and guidance in treatment measures [106,107]. Initial steps of most treatment algorithms include SLTs, obtaining patient history (e.g., usage of prescribed oral anticoagulants), and basics of coagulation management, i.e., initial administration of tranexamic acid (TXA) in line with current guidelines [14], followed by fibrinogen administration [107]. Fibrinogen administration as well as the following steps are often based on results of VET testing, which is not available in all hospitals, and there is little experience or data on concentrate-based management without it [107]. SLTs have a long turn-around time—the standard Clauss fibrinogen test takes on average 40–45 min; therefore, the assessment of plasma fibrinogen levels within the first hour of admission without VET is challenging [108]. However, some parameters allow the estimation of plasma fibrinogen levels based on SLTs and enable rapid FC supplementation even without VET [107]. These can be implemented into alternative coagulation management guidelines (Figure 2) [107].

The Fibrinogen on Admission in Trauma (FibAT) score was developed as a simple clinical tool to predict low fibrinogen concentration in trauma patients on arrival at the hospital [109]. Independent predictive factors for low plasma fibrinogen levels were identified by a multivariate logistic regression model and included factors such as age, heart rate, body temperature, haemoglobin (Hb) concentration on admission, and blood lactate level [109]. A final FibAT score of 5 or above was able to reliably predict trauma patients with fibrinogen levels at 1.5 g/L or below on admission [109]. Another immediate physiological assessment of haemorrhagic shock in trauma patients is provided by base-deficit (BD) measurement that was found to be a better mortality predictor than vital signs [110]. Another study showed that fibrinogen levels show strong correlation with rapidly obtainable routine laboratory parameters, such as base excess (BE) and Hb [108]. These two parameters might provide a sensitive and quick tool to identify major trauma patients that are at risk of reaching critically low fibrinogen. Both parameters are now included in the European Guidelines for bleeding following trauma [14]. Initial Hb is used as indicator for severe bleeding, associated with coagulopathy (Grade 1B) and BD measurements to estimate the extent of bleeding (Grade 1B) [14].

**Figure 3:**
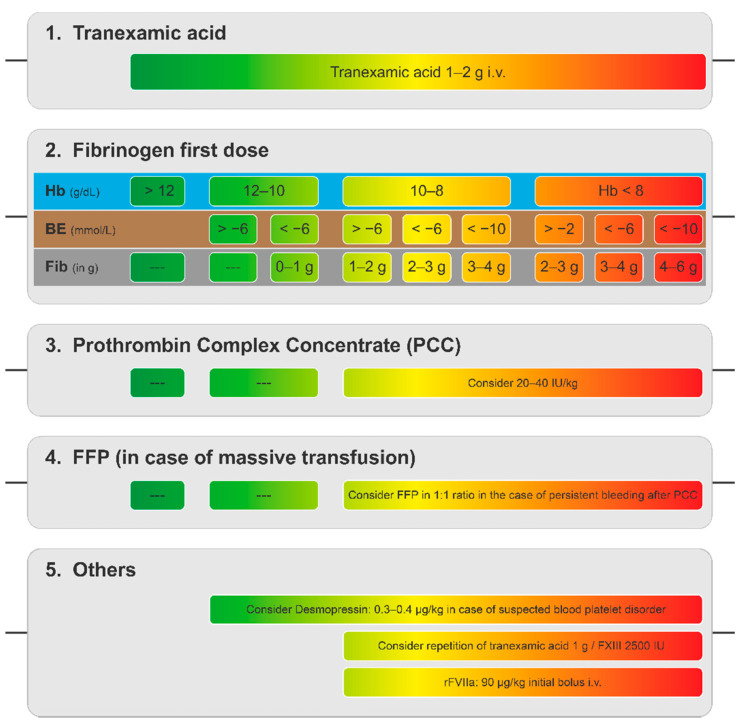
Decision tree for dosing of haemostatic agents without the use of VET. Adapted from Casu S, 2021 [107]. Reprinted by permission from BMJ as indicated in the Terms and Conditions of the Creative Commons CC-NY license. BE, base excess; Fib, fibrinogen; FFP, fresh frozen plasma; Hb, haemoglobin; i.v., intravenously; rFVIIa, activated recombinant factor VII.

These findings were integrated into a decision tree (Figure 3) to guide fibrinogen dosing based on Hb and BE analysis as these parameters consider the extent of the trauma instead of blindly estimating the required dose [107]. These results are routinely available within minutes as part of blood gas analysis, and can serve as an effective alternative to conventional POC testing [107]. In this way, the published guidelines can be adapted to the local resources and help provide efficient GDCM of trauma-associated bleeding. Limitation of this protocol adaptation is that it is still blind in many aspects, and further studies will be required to validate this method.

## 7. Conclusions

Despite general improvements in the management of bleeding patients, the treatment protocols vary considerably in different places. This is not only due to local differences in available resources but also a lack of solid evidence supporting one protocol over another. The aim of GDCM is a tailored haemostatic therapy for each patient that would provide the most suitable treatment in the given situation. Algorithms based on VET allow for individualised treatment and reduce the risks of over- or under-transfusion. Moreover, CFCs are an effective and promising alternative to ratio-based transfusion therapies for the correction of different types of coagulopathies and are increasingly incorporated as the first-line treatment option into many guidelines. Finally, initial concentrate-based management is possible also without POC testing; dosage can be also based on parameters from blood gas analysis, offering a vital alternative to ensure patients receive the optimal care.

## Figures and Tables

**Table 1:** Summary of guideline recommendations for the treatment of bleeding related to VKA and DOAC.

Organisation	Recommendation	Grade
A. Recommendations for the treatment of VKA-associated bleeding		
American College of Cardiology [46]	4F-PCC (FFP only if PCC not available)	
American College of Chest Physicians [44]	vit K + 4F-PCC over plasma	2C
Neurocritical Care Society [47]	vit K + 4F-PCC over FFP	strong recommendation, moderate quality
American Society for Gastrointestinal Endoscopy [48]	vit K + 4F-PCC over FFP	moderate quality
American Society of Anesthesiologists [49]	vit K + 4F-PCC or FFP	
American Society of Hematology [50]	vit K + 4F PCC over FFP	conditional recommendation
American College of Emergency Physicians [51]	vit K + 4F-PCC over FFP	
Canadian stroke best practice recommendations [52]	vit K + PCC	evidence level B
European Guidelines [14]	vit K + PCC	1A
European Society of Anaesthesiology [12]	vit K + PCC	1B
British Committee for Standards in Haematology [53]	vit K + 4F-PCC (FFP only if PCC not available)	1B
French clinical practice [45]	vit K + PCC (FFP only if PCC not available)	
European Society of Gastrointestinal Endoscopy [42]	vit K + PCC (FFP if PCC not available)	strong recommendation, low quality evidence
European Stroke Organisation [41]	vit K + PCC over FFP	strong recommendation, moderate quality evidence
European Association of Cardiothoracic Anaesthesiology [54]	4F-PCC	
B. Recommendations for the treatment of DOAC-associated bleeding		
American College of Cardiology [46]	FXa-i: 1st line andexanet alfa, 2nd line PCC/aPCC	
	FIIa: 1st line idarucizumab, 2nd line PCC/aPCC	
American Society for Gastrointestinal Endoscopy [48]	FXa-i: PCC/aPCC	
	FIIa: PCC/aPCC	
American Society of Hematology [50]	FXa-i: 4F-PCC or andexanet alfa	conditional recommendation
	FIIa: idarucizumab	
American College of Emergency Physicians [51]	FXa-i: 1st line andexanet alfa, 2nd line 4F-PCC	
	FIIa: 1st line idarucizumab, 2nd line 4F-PCC over 3F-PCC	
Canadian stroke best practice recommendations [52]	FXa-i: PCC	evidence level C
	FIIa: 1st line idarucizumab, 2nd line PCC/aPCC	
European Guidelines [14]	FXa-i: TXA and PCC, until specific antidotes available	2C
	FIIa: idarucizumab	1B
European Society of Anaesthesiology [12]	FXa-i: N/A	2C
	FIIa: idarucizumab	
European Society of Gastrointestinal Endoscopy [42]	DOAC reversal agent or PCC	strong recommendation, low quality evidence
European Stroke Organisation [41]	4F-PCC, if specific reversal agents not available	weak recommendation, very low-quality evidence
European Association of Cardiothoracic Anaesthesiology [54]	4F-PCC, if specific reversal agents not available	–

3F, three-factor; 4F, four-factor; aPCC, activated prothrombin complex concentrate; DOAC, direct oral anticoagulant; FIIa, activated factor II (thrombin) inhibitor; FFP, fresh frozen plasma; FXa-i, activated factor X inhibitor; N/A, not available; PCC, prothrombin complex concentrate; TXA, tranexamic acid; vit K, vitamin K; VKA, vitamin K antagonists.

**Table 2:** Comparison of standard laboratory tests vs. point-of-care testing [79,81,82].

	Standard Laboratory Tests	Point-of-Care Testing
Tests	Platelet count, INR, PT, aPTT, Clauss fibrinogen	Viscoelastic testing: TEG, TEM
Advantages	Higher accuracy Lower cost	Quick turn-around: preliminary results in 5 min, full results in 10–20 min Uses whole blood, allows for interaction of all the clot components: platelet, factors, RBCs Provides feedback on the treatment, enables stepwise management
Disadvantages	Poor predictors of bleeding Long turnaround time (ca 60 min) Do not provide information about dynamic changes Performed at normal pH/temperature (might not reflect in vivo situation, i.e., hypothermia, acidosis)	Quality standards are lower than in laboratory tests Less precise Requires training of the staff More expensive

aPTT, activated partial thromboplastin time; INR, international normalised ratio; PT, prothrombin time; RBCs, red blood cells; TEG, thrombelastography; TEM, thromboelastometry.

## Data Availability

The data are available from the online database or on request from the corresponding author.

## References

[1] Hurwitz A., Massone R., Lopez B.L. (2014). Acquired bleeding disorders. Emerg. Med. Clin. N. Am..

[2] Grottke O., Fries D., Nascimento B. (2015). Perioperatively acquired disorders of coagulation. Curr. Opin. Anaesthesiol..

[3] Bolliger D., Görlinger K., Tanaka K.A. (2010). Pathophysiology and treatment of coagulopathy in massive hemorrhage and hemodilution. Anesthesiology.

[4] Tanaka K., Bolliger D. (2014). Acquired coagulopathy. Reference Module in Biomedical Sciences.

[5] Hardy J.F., De Moerloose P., Samama M. (2004). Massive transfusion and coagulopathy: Pathophysiology and implications for clinical management. Can. J. Anaesth..

[6] Schochl H., Maegele M., Solomon C., Gorlinger K., Voelckel W. (2012). Early and individualized goal-directed therapy for trauma-induced coagulopathy. Scand. J. Trauma Resusc. Emerg. Med..

[7] Hiippala S.T., Myllylä G.J., Vahtera E.M. (1995). Hemostatic factors and replacement of major blood loss with plasma-poor red cell concentrates. Anesth. Analg..

[8] Litvinov R.I., Pieters M., de Lange-Loots Z., Weisel J.W. (2021). Fibrinogen and fibrin. Subcell. Biochem..

[9] Lowe G.D., Rumley A., Woodward M., Morrison C.E., Philippou H., Lane D.A., Tunstall-Pedoe H. (1997). Epidemiology of coagulation factors, inhibitors and activation markers: The Third Glasgow MONICA Survey. I. Illustrative reference ranges by age, sex and hormone use. Br. J. Haematol..

[10] Fenger-Eriksen C., Ingerslev J., Sorensen B. (2009). Fibrinogen concentrate--a potential universal hemostatic agent. Expert Opin. Biol. Ther..

[11] Kreuz W., Meili E., Peter-Salonen K., Haertel S., Devay J., Krzensk U., Egbring R. (2005). Efficacy and tolerability of a pasteurised human fibrinogen concentrate in patients with congenital fibrinogen deficiency. Transfus. Apher. Sci..

[12] Kozek-Langenecker S.A., Ahmed A.B., Afshari A., Albaladejo P., Aldecoa C., Barauskas G., De Robertis E., Faraoni D., Filipescu D.C., Fries D. (2017). Management of severe perioperative bleeding: Guidelines from the European Society of Anaesthesiology: First update 2016. Eur. J. Anaesthesiol..

[13] McQuilten Z.K., Bailey M., Cameron P.A., Stanworth S.J., Venardos K., Wood E.M., Cooper D.J. (2017). Fibrinogen concentration and use of fibrinogen supplementation with cryoprecipitate in patients with critical bleeding receiving massive transfusion: A bi-national cohort study. Br. J. Haematol..

[14] Spahn D.R., Bouillon B., Cerny V., Duranteau J., Filipescu D., Hunt B.J., Komadina R., Maegele M., Nardi G., Riddez L. (2019). The European guideline on management of major bleeding and coagulopathy following trauma: Fifth edition. Crit. Care.

[15] Cerneca F., Ricci G., Simeone R., Malisano M., Alberico S., Guaschino S. (1997). Coagulation and fibrinolysis changes in normal pregnancy. Increased levels of procoagulants and reduced levels of inhibitors during pregnancy induce a hypercoagulable state, combined with a reactive fibrinolysis. Eur. J. Obstet. Gynecol. Reprod. Biol..

[16] Bremme K.A. (2003). Haemostatic changes in pregnancy. Best Pract. Res. Clin. Haematol..

[17] Collins P.W., Lilley G., Bruynseels D., Laurent D.B., Cannings-John R., Precious E., Hamlyn V., Sanders J., Alikhan R., Rayment R. (2014). Fibrin-based clot formation as an early and rapid biomarker for progression of postpartum hemorrhage: A prospective study. Blood.

[18] Charbit B., Mandelbrot L., Samain E., Baron G., Haddaoui B., Keita H., Sibony O., Mahieu-Caputo D., Hurtaud-Roux M.F., Huisse M.G. (2007). The decrease of fibrinogen is an early predictor of the severity of postpartum hemorrhage. J. Thromb. Haemost..

[19] Patil V., Shetmahajan M. (2014). Massive transfusion and massive transfusion protocol. Indian J. Anaesth..

[20] Theusinger O.M., Baulig W., Seifert B., Emmert M.Y., Spahn D.R., Asmis L.M. (2011). Relative concentrations of haemostatic factors and cytokines in solvent/detergent-treated and fresh-frozen plasma. Br. J. Anaesth..

[21] Tanaka K.A., Esper S., Bolliger D. (2013). Perioperative factor concentrate therapy. Br. J. Anaesth..

[22] Collins P.W., Solomon C., Sutor K., Crispin D., Hochleitner G., Rizoli S., Schochl H., Schreiber M., Ranucci M. (2014). Theoretical modelling of fibrinogen supplementation with therapeutic plasma, cryoprecipitate, or fibrinogen concentrate. Br. J. Anaesth..

[23] Kozek-Langenecker S., Sørensen B., Hess J.R., Spahn D.R. (2011). Clinical effectiveness of fresh frozen plasma compared with fibrinogen concentrate: A systematic review. Crit. Care.

[24] Khan S., Brohi K., Chana M., Raza I., Stanworth S., Gaarder C., Davenport R. (2014). Hemostatic resuscitation is neither hemostatic nor resuscitative in trauma hemorrhage. J. Trauma Acute Care Surg..

[25] Innerhofer P., Fries D., Mittermayr M., Innerhofer N., von Langen D., Hell T., Gruber G., Schmid S., Friesenecker B., Lorenz I.H. (2017). Reversal of trauma-induced coagulopathy using first-line coagulation factor concentrates or fresh frozen plasma (RETIC): A single-centre, parallel-group, open-label, randomised trial. Lancet Haematol..

[26] Morrison G.A., Koch J., Royds M., McGee D., Chalmers R.T.A., Anderson J., Nimmo A.F. (2019). Fibrinogen concentrate vs. fresh frozen plasma for the management of coagulopathy during thoraco-abdominal aortic aneurysm surgery: A pilot randomised controlled trial. Anaesthesia.

[27] Weitz J.I., Eikelboom J.W., Samama M.M. (2012). New antithrombotic drugs: Antithrombotic therapy and prevention of thrombosis, 9th ed: American College of Chest Physicians Evidence-Based Clinical Practice Guidelines. Chest.

[28] Loo S.Y., Dell’Aniello S., Huiart L., Renoux C. (2017). Trends in the prescription of novel oral anticoagulants in UK primary care. Br. J. Clin. Pharmacol..

[29] Ho K.H., van Hove M., Leng G. (2020). Trends in anticoagulant prescribing: A review of local policies in English primary care. BMC Health Serv. Res..

[30] Quinlan D.J., Eikelboom J.W., Weitz J.I. (2013). Four-factor prothrombin complex concentrate for urgent reversal of vitamin K antagonists in patients with major bleeding. Circulation.

[31] Watson H.G., Baglin T., Laidlaw S.L., Makris M., Preston F.E. (2001). A comparison of the efficacy and rate of response to oral and intravenous Vitamin K in reversal of over-anticoagulation with warfarin. Br. J. Haematol..

[32] Narick C., Triulzi D.J., Yazer M.H. (2012). Transfusion-associated circulatory overload after plasma transfusion. Transfusion.

[33] Franchini M., Lippi G. (2010). Prothrombin complex concentrates: An update. Blood Transfus..

[34] Brekelmans M.P.A., Ginkel K.V., Daams J.G., Hutten B.A., Middeldorp S., Coppens M. (2017). Benefits and harms of 4-factor prothrombin complex concentrate for reversal of vitamin K antagonist associated bleeding: A systematic review and meta-analysis. J. Thromb. Thrombolysis.

[35] Chai-Adisaksopha C., Hillis C., Siegal D.M., Movilla R., Heddle N., Iorio A., Crowther M. (2016). Prothrombin complex concentrates versus fresh frozen plasma for warfarin reversal. A systematic review and meta-analysis. Thromb. Haemost..

[36] Hill R., Han T.S., Lubomirova I., Math N., Bentley P., Sharma P. (2019). Prothrombin complex concentrates are superior to fresh frozen plasma for emergency reversal of vitamin K antagonists: A meta-analysis in 2606 subjects. Drugs.

[37] Zapata-Wainberg G., Ximénez-Carrillo Rico Á., Benavente Fernández L., Masjuan Vallejo J., Gállego Culleré J., Freijó Guerrero M.D.M., Egido J., Gómez Sánchez J.C., Martínez Domeño A., Purroy García F. (2015). Epidemiology of intracranial haemorrhages associated with vitamin K antagonist oral anticoagulants in Spain: TAC Registry. Interv. Neurol..

[38] Flibotte J.J., Hagan N., O’Donnell J., Greenberg S.M., Rosand J. (2004). Warfarin, hematoma expansion, and outcome of intracerebral hemorrhage. Neurology.

[39] Parry-Jones A.R., Di Napoli M., Goldstein J.N., Schreuder F.H., Tetri S., Tatlisumak T., Yan B., van Nieuwenhuizen K.M., Dequatre-Ponchelle N., Lee-Archer M. (2015). Reversal strategies for vitamin K antagonists in acute intracerebral hemorrhage. Ann. Neurol..

[40] Steiner T., Poli S., Griebe M., Hüsing J., Hajda J., Freiberger A., Bendszus M., Bösel J., Christensen H., Dohmen C. (2016). Fresh frozen plasma versus prothrombin complex concentrate in patients with intracranial haemorrhage related to vitamin K antagonists (INCH): A randomised trial. Lancet Neurol..

[41] Christensen H., Cordonnier C., Korv J., Lal A., Ovesen C., Purrucker J.C., Toni D., Steiner T. (2019). European Stroke Organisation guideline on reversal of oral anticoagulants in acute intracerebral haemorrhage. Eur. Stroke J..

[42] Gralnek I.M., Stanley A.J., Morris A.J., Camus M., Lau J., Lanas A., Laursen S.B., Radaelli F., Papanikolaou I.S., Curdia Goncalves T. (2021). Endoscopic diagnosis and management of nonvariceal upper gastrointestinal hemorrhage (NVUGIH): European Society of Gastrointestinal Endoscopy (ESGE) Guideline—Update 2021. Endoscopy.

[43] Maegele M. (2021). The European perspective on the management of acute major hemorrhage and coagulopathy after trauma: Summary of the 2019 updated European guideline. J. Clin. Med..

[44] Holbrook A., Schulman S., Witt D.M., Vandvik P.O., Fish J., Kovacs M.J., Svensson P.J., Veenstra D.L., Crowther M., Guyatt G.H. (2012). Evidence-based management of anticoagulant therapy: Antithrombotic therapy and prevention of thrombosis, 9th ed: American College of Chest Physicians evidence-based clinical practice guidelines. Chest.

[45] Pernod G., Godier A., Gozalo C., Tremey B., Sie P., French National Authority for Haematology (2010). French clinical practice guidelines on the management of patients on vitamin K antagonists in at-risk situations (overdose, risk of bleeding, and active bleeding). Thromb. Res..

[46] Tomaselli G.F., Mahaffey K.W., Cuker A., Dobesh P.P., Doherty J.U., Eikelboom J.W., Florido R., Gluckman T.J., Hucker W.J., Mehran R. (2020). 2020 ACC expert consensus decision pathway on management of bleeding in patients on oral anticoagulants: A report of the American College of Cardiology Solution Set Oversight Committee. J. Am. Coll. Cardiol..

[47] Frontera J.A., Lewin J.J., Rabinstein A.A., Aisiku I.P., Alexandrov A.W., Cook A.M., del Zoppo G.J., Kumar M.A., Peerschke E.I., Stiefel M.F. (2016). Guideline for reversal of antithrombotics in intracranial hemorrhage: A statement for healthcare professionals from the Neurocritical Care Society and Society of Critical Care Medicine. Neurocrit. Care.

[48] Acosta R.D., Abraham N.S., Chandrasekhara V., Chathadi K.V., Early D.S., Eloubeidi M.A., Evans J.A., Faulx A.L., Fisher D.A., Fonkalsrud L. (2016). The management of antithrombotic agents for patients undergoing GI endoscopy. Gastrointest. Endosc..

[49] ASA (2015). Practice guidelines for perioperative blood management: An updated report by the American Society of Anesthesiologists Task Force on Perioperative Blood Management. Anesthesiology.

[50] Witt D.M., Nieuwlaat R., Clark N.P., Ansell J., Holbrook A., Skov J., Shehab N., Mock J., Myers T., Dentali F. (2018). American Society of Hematology 2018 guidelines for management of venous thromboembolism: Optimal management of anticoagulation therapy. Blood Adv..

[51] Baugh C.W., Levine M., Cornutt D., Wilson J.W., Kwun R., Mahan C.E., Pollack C.V., Marcolini E.G., Milling T.J., Peacock W.F. (2020). Anticoagulant reversal strategies in the emergency department setting: Recommendations of a multidisciplinary expert panel. Ann. Emerg. Med..

[52] Shoamanesh A., Patrice Lindsay M., Castellucci L.A., Cayley A., Crowther M., de Wit K., English S.W., Hoosein S., Huynh T., Kelly M. (2021). Canadian stroke best practice recommendations: Management of spontaneous intracerebral hemorrhage, 7th Edition Update 2020. Int. J. Stroke.

[53] Keeling D., Baglin T., Tait C., Watson H., Perry D., Baglin C., Kitchen S., Makris M., British Committee for Standards in Haematology (2011). Guidelines on oral anticoagulation with warfarin—Fourth edition. Br. J. Haematol..

[54] Erdoes G., Koster A., Ortmann E., Meesters M.I., Bolliger D., Baryshnikova E., Martinez Lopez De Arroyabe B., Ahmed A., Lance M.D., Ranucci M. (2021). A European consensus statement on the use of four-factor prothrombin complex concentrate for cardiac and non-cardiac surgical patients. Anaesthesia.

[55] Marano G., Vaglio S., Pupella S., Liumbruno G.M., Franchini M. (2016). How we treat bleeding associated with direct oral anticoagulants. Blood Transfus..

[56] Kustos S.A., Fasinu P.S. (2019). Direct-acting oral anticoagulants and their reversal agents-An update. Medicines.

[57] Grottke O., Schulman S. (2019). Four-factor prothrombin complex concentrate for the management of patients receiving direct oral activated factor X inhibitors. Anesthesiology.

[58] Cuker A., Burnett A., Triller D., Crowther M., Ansell J., Van Cott E.M., Wirth D., Kaatz S. (2019). Reversal of direct oral anticoagulants: Guidance from the Anticoagulation Forum. Am. J. Hematol..

[59] Hoffman M., Goldstein J.N., Levy J.H. (2018). The impact of prothrombin complex concentrates when treating DOAC-associated bleeding: A review. Int. J. Emerg. Med..

[60] Tomaselli G.F., Mahaffey K.W., Cuker A., Dobesh P.P., Doherty J.U., Eikelboom J.W., Florido R., Hucker W., Mehran R., Messé S.R. (2017). 2017 ACC expert consensus decision pathway on management of bleeding in patients on oral anticoagulants: A report of the American College of Cardiology Task Force on expert consensus decision pathways. J. Am. Coll. Cardiol..

[61] Schulman S., Gross P.L., Ritchie B., Nahirniak S., Lin Y., Lieberman L., Carrier M., Crowther M.A., Ghosh I., Lazo-Langner A. (2018). Prothrombin complex concentrate for major bleeding on factor Xa inhibitors: A prospective cohort study. Thromb. Haemost..

[62] Majeed A., Ågren A., Holmström M., Bruzelius M., Chaireti R., Odeberg J., Hempel E.L., Magnusson M., Frisk T., Schulman S. (2017). Management of rivaroxaban- or apixaban-associated major bleeding with prothrombin complex concentrates: A cohort study. Blood.

[63] Lipari L., Yang S., Milligan B., Blunck J. (2020). Emergent reversal of oral factor Xa inhibitors with four-factor prothrombin complex concentrate. Am. J. Emerg. Med..

[64] Connolly S.J., Crowther M., Eikelboom J.W., Gibson C.M., Curnutte J.T., Lawrence J.H., Yue P., Bronson M.D., Lu G., Conley P.B. (2019). Full study report of andexanet alfa for bleeding associated with factor Xa inhibitors. N. Engl. J. Med..

[65] Allison T.A., Lin P.J., Gass J.A., Chong K., Prater S.J., Escobar M.A., Hartman H.D. (2020). Evaluation of the use of low-dose 4-factor prothrombin complex concentrate in the reversal of direct oral anticoagulants in bleeding patients. J. Intensive Care Med..

[66] Berger K., Santibañez M., Lin L., Lesch C.A. (2020). A low-dose 4F-PCC protocol for DOAC-associated intracranial hemorrhage. J. Intensive Care Med..

[67] Ammar A.A., Ammar M.A., Owusu K.A., Brown S.C., Kaddouh F., Elsamadicy A.A., Acosta J.N., Falcone G.J. (2021). Andexanet alfa versus 4-factor prothrombin complex concentrate for reversal of factor Xa inhibitors in intracranial hemorrhage. Neurocrit. Care.

[68] Barra M.E., Das A.S., Hayes B.D., Rosenthal E.S., Rosovsky R.P., Fuh L., Patel A.B., Goldstein J.N., Roberts R.J. (2020). Evaluation of andexanet alfa and four-factor prothrombin complex concentrate (4F-PCC) for reversal of rivaroxaban- and apixaban-associated intracranial hemorrhages. J. Thromb. Haemost..

[69] Frontera J.A., Bhatt P., Lalchan R., Yaghi S., Ahuja T., Papadopoulos J., Joset D. (2020). Cost comparison of andexanet versus prothrombin complex concentrates for direct factor Xa inhibitor reversal after hemorrhage. J. Thromb. Thrombolysis.

[70] Costa O.S., Baker W.L., Roman-Morillo Y., McNeil-Posey K., Lovelace B., White C.M., Coleman C.I. (2020). Quality evaluation of case series describing four-factor prothrombin complex concentrate in oral factor Xa inhibitor-associated bleeding: A systematic review. BMJ Open.

[71] Luo C., Chen F., Chen Y.H., Zhao C.F., Feng C.Z., Liu H.X., Luo D.Z.Q. (2021). Prothrombin complex concentrates and andexanet for management of direct factor Xa inhibitor related bleeding: A meta-analysis. Eur. Rev. Med. Pharmacol. Sci..

[72] Nederpelt C.J., Naar L., Krijnen P., le Cessie S., Kaafarani H.M.A., Huisman M.V., Velmahos G.C., Schipper I.B. (2021). Andexanet alfa or prothrombin complex concentrate for factor Xa inhibitor reversal in acute major bleeding: A systematic review and meta-analysis. Crit. Care Med..

[73] Jaspers T., Shudofsky K., Huisman M.V., Meijer K., Khorsand N. (2021). A meta-analysis of andexanet alfa and prothrombin complex concentrate in the treatment of factor Xa inhibitor-related major bleeding. Res. Pract. Thromb. Haemost..

[74] Moore E.E., Moore H.B., Kornblith L.Z., Neal M.D., Hoffman M., Mutch N.J., Schöchl H., Hunt B.J., Sauaia A. (2021). Trauma-induced coagulopathy. Nat. Rev. Dis. Primers.

[75] Brohi K., Singh J., Heron M., Coats T. (2003). Acute traumatic coagulopathy. J. Trauma Acute Care Surg..

[76] Floccard B., Rugeri L., Faure A., Saint Denis M., Boyle E.M., Peguet O., Levrat A., Guillaume C., Marcotte G., Vulliez A. (2012). Early coagulopathy in trauma patients: An on-scene and hospital admission study. Injury.

[77] Khan S., Davenport R., Raza I., Glasgow S., De’Ath H.D., Johansson P.I., Curry N., Stanworth S., Gaarder C., Brohi K. (2015). Damage control resuscitation using blood component therapy in standard doses has a limited effect on coagulopathy during trauma hemorrhage. Intensive Care Med..

[78] Shaz B.H., Winkler A.M., James A.B., Hillyer C.D., MacLeod J.B. (2011). Pathophysiology of early trauma-induced coagulopathy: Emerging evidence for hemodilution and coagulation factor depletion. J. Trauma.

[79] Haas T., Fries D., Tanaka K.A., Asmis L., Curry N.S., Schöchl H. (2015). Usefulness of standard plasma coagulation tests in the management of perioperative coagulopathic bleeding: Is there any evidence?. Br. J. Anaesth..

[80] Schochl H., Voelckel W., Schlimp C.J. (2015). Management of traumatic haemorrhage—The European perspective. Anaesthesia.

[81] Gonzalez E., Moore E.E., Moore H.B., Chapman M.P., Chin T.L., Ghasabyan A., Wohlauer M.V., Barnett C.C., Bensard D.D., Biffl W.L. (2016). Goal-directed hemostatic resuscitation of trauma-induced coagulopathy: A pragmatic randomized clinical trial comparing a viscoelastic assay to conventional coagulation assays. Ann. Surg..

[82] Wool G.D. (2018). Benefits and pitfalls of point-of-care coagulation testing for anticoagulation management: An ACLPS critical review. Am. J. Clin. Pathol..

[83] Geeraedts L.M., Demiral H., Schaap N.P., Kamphuisen P.W., Pompe J.C., Frolke J.P. (2007). ‘Blind’ transfusion of blood products in exsanguinating trauma patients. Resuscitation.

[84] Ponschab M., Schochl H., Gabriel C., Sussner S., Cadamuro J., Haschke-Becher E., Gratz J., Zipperle J., Redl H., Schlimp C.J. (2015). Haemostatic profile of reconstituted blood in a proposed 1:1:1 ratio of packed red blood cells, platelet concentrate and four different plasma preparations. Anaesthesia.

[85] Desborough M., Sandu R., Brunskill S.J., Doree C., Trivella M., Montedori A., Abraha I., Stanworth S. (2015). Fresh frozen plasma for cardiovascular surgery. Cochrane Database Syst. Rev..

[86] Schöchl H., Nienaber U., Hofer G., Voelckel W., Jambor C., Scharbert G., Kozek-Langenecker S., Solomon C. (2010). Goal-directed coagulation management of major trauma patients using thromboelastometry (ROTEM)-guided administration of fibrinogen concentrate and prothrombin complex concentrate. Crit. Care.

[87] Yang L., Stanworth S., Hopewell S., Doree C., Murphy M. (2012). Is fresh-frozen plasma clinically effective? An update of a systematic review of randomized controlled trials. Transfusion.

[88] Stein P., Kaserer A., Sprengel K., Wanner G.A., Seifert B., Theusinger O.M., Spahn D.R. (2017). Change of transfusion and treatment paradigm in major trauma patients. Anaesthesia.

[89] Nardi G., Agostini V., Rondinelli B., Russo E., Bastianini B., Bini G., Bulgarelli S., Cingolani E., Donato A., Gambale G. (2015). Trauma-induced coagulopathy: Impact of the early coagulation support protocol on blood product consumption, mortality and costs. Crit. Care.

[90] Hagemo J.S., Stanworth S., Juffermans N.P., Brohi K., Cohen M., Johansson P.I., Røislien J., Eken T., Næss P.A., Gaarder C. (2014). Prevalence, predictors and outcome of hypofibrinogenaemia in trauma: A multicentre observational study. Crit. Care.

[91] Schlimp C.J., Ponschab M., Voelckel W., Treichl B., Maegele M., Schochl H. (2016). Fibrinogen levels in trauma patients during the first seven days after fibrinogen concentrate therapy: A retrospective study. Scand. J. Trauma Resusc. Emerg. Med..

[92] Schöchl H., Cotton B., Inaba K., Nienaber U., Fischer H., Voelckel W., Solomon C. (2011). FIBTEM provides early prediction of massive transfusion in trauma. Crit. Care.

[93] Meyer M.A.S., Ostrowski S.R., Sørensen A.M., Meyer A.S.P., Holcomb J.B., Wade C.E., Johansson P.I., Stensballe J. (2015). Fibrinogen in trauma, an evaluation of thrombelastography and rotational thromboelastometry fibrinogen assays. J. Surg. Res..

[94] Erdoes G., Gerster G., Colucci G., Kaiser H., Alberio L., Eberle B. (2015). Prediction of post-weaning fibrinogen status during cardiopulmonary bypass: An observational study in 110 patients. PLoS ONE.

[95] Nascimento B., Callum J., Tien H., Peng H., Rizoli S., Karanicolas P., Alam A., Xiong W., Selby R., Garzon A.M. (2016). Fibrinogen in the initial resuscitation of severe trauma (FiiRST): A randomized feasibility trial. Br. J. Anaesth..

[96] Itagaki Y., Hayakawa M., Maekawa K., Saito T., Kodate A., Honma Y., Mizugaki A., Yoshida T., Ohyasu T., Katabami K. (2020). Early administration of fibrinogen concentrate is associated with improved survival among severe trauma patients: A single-centre propensity score-matched analysis. World J. Emerg. Surg..

[97] Gratz J., Schlimp C.J., Honickel M., Hochhausen N., Schöchl H., Grottke O. (2020). Sufficient thrombin generation despite 95% hemodilution: An iIn vitro experimental study. J. Clin. Med..

[98] Dunbar N.M., Chandler W.L. (2009). TRANSFUSION PRACTICE: Thrombin generation in trauma patients. Transfusion.

[99] Ponschab M., Voelckel W., Pavelka M., Schlimp C.J., Schöchl H. (2015). Effect of coagulation factor concentrate administration on ROTEM® parameters in major trauma. Scand. J. Trauma Resusc. Emerg. Med..

[100] Schochl H., Voelckel W., Maegele M., Kirchmair L., Schlimp C.J. (2014). Endogenous thrombin potential following hemostatic therapy with 4-factor prothrombin complex concentrate: A 7-day observational study of trauma patients. Crit. Care.

[101] Hess J.R., Lindell A.L., Stansbury L.G., Dutton R.P., Scalea T.M. (2009). The prevalence of abnormal results of conventional coagulation tests on admission to a trauma center. Transfusion.

[102] Muszbek L., Adány R., Mikkola H. (1996). Novel aspects of blood coagulation factor XIII. I. Structure, distribution, activation, and function. Crit. Rev. Clin. Lab. Sci..

[103] Raspé C., Besch M., Charitos E.I., Flöther L., Bucher M., Rückert F., Treede H. (2018). Rotational thromboelastometry for assessing bleeding complications and factor XIII deficiency in cardiac surgery patients. Clin. Appl. Thromb. Hemost..

[104] Bedreli S., Sowa J.P., Malek S., Blomeyer S., Katsounas A., Gerken G., Saner F.H., Canbay A. (2017). Rotational thromboelastometry can detect factor XIII deficiency and bleeding diathesis in patients with cirrhosis. Liver Int..

[105] Gerlach R., Raabe A., Zimmermann M., Siegemund A., Seifert V. (2000). Factor XIII deficiency and postoperative hemorrhage after neurosurgical procedures. Surg. Neurol..

[106] Maegele M. (2019). The Diagnosis and treatment of acute traumatic bleeding and coagulopathy. Dtsch. Arztebl. Int..

[107] Casu S. (2021). Simplified treatment algorithm for the management of trauma-induced hemorrhage without viscoelastic testing. Trauma Surg. Acute Care Open.

[108] Schlimp C.J., Voelckel W., Inaba K., Maegele M., Ponschab M., Schochl H. (2013). Estimation of plasma fibrinogen levels based on hemoglobin, base excess and Injury Severity Score upon emergency room admission. Crit. Care.

[109] Gauss T., Campion S., Kerever S., Eurin M., Raux M., Harrois A., Paugam-Burtz C., Hamada S., Traumabase G. (2018). Fibrinogen on Admission in Trauma score: Early prediction of low plasma fibrinogen concentrations in trauma patients. Eur. J. Anaesthesiol..

[110] Dunham M.P., Sartorius B., Laing G.L., Bruce J.L., Clarke D.L. (2017). A comparison of base deficit and vital signs in the early assessment of patients with penetrating trauma in a high burden setting. Injury.

